# Lumpy Skin Disease Virus Pathogenesis: Viral Protein Functions and Comparative Insights from Vaccinia Virus

**DOI:** 10.3390/ani15213176

**Published:** 2025-10-31

**Authors:** Huan Chen, Ruiyu Zhai, Chang Cai, Xiaojie Zhu, Yong-Sam Jung, Yingjuan Qian

**Affiliations:** 1 Sanya Institute of Nanjing Agricultural University, Laboratory of Emerging Animal Diseases and One Health, Nanjing Agricultural University, Nanjing 210095, China; chenhuan2019@njau.edu.cn (H.C.); zhairy2022@163.com (R.Z.); caichang116@hotmail.com (C.C.); 2 MOE Joint International Research Laboratory of Animal Health and Food Safety, College of Veterinary Medicine, Nanjing Agricultural University, Nanjing 210095, China; 3 China Institute of Veterinary Drug Control, Beijing 100081, China; zhuxiaojie0603@163.com

**Keywords:** *lumpy skin disease virus* (LSDV), viral proteins, viral life cycle, immune evasion, comparative analysis

## Abstract

**Simple Summary:**

*Lumpy skin disease* is a significant viral infection affecting cattle, and it is characterized by skin nodules, fever, and potentially fatal outcomes, all of which contribute to substantial economic losses in the livestock industry. This review synthesizes existing understanding of the molecular mechanisms employed by the *Lumpy Skin Disease Virus* (LSDV), focusing on its ability to enter host cells, replicate, and evade immune defenses. Through comparative analysis with *vaccinia virus*, a well-characterized relative, we elucidate both conserved and unique strategies that enhance viral survival and transmission. The findings demonstrate that LSDV encodes multiple proteins capable of disrupting host immunity, thereby promoting infection. These insights deepen our comprehension of viral pathogenesis and provide a foundation for developing improved vaccines and control measures aimed at mitigating outbreaks, enhancing animal health, and supporting sustainable agricultural economies.

**Abstract:**

*Lumpy Skin Disease Virus* (LSDV), a member of the poxvirus family, represents a significant threat to global cattle industries. This review presents an analysis of LSDV-encoded proteins and their interactions with host systems, elucidating the molecular mechanisms governing viral life cycle progression and immune evasion strategies. We provide detailed characterization of the complex architecture of LSDV virions, including Intracellular Mature Virus (IMV), Extracellular Enveloped Virus (EEV), lateral bodies, and the core components, while summarizing the crucial functions of viral proteins throughout various stages of infection—entry, replication, transcription, translation, assembly, and egress. Particular attention is given to the immunomodulatory strategies employed by LSDV to subvert both innate and adaptive immune responses. These mechanisms encompass molecular mimicry of cytokines and chemokines, interference with antigen presentation pathways, inhibition of key immune signaling cascades, and modulation of apoptosis and autophagy processes. Through comparative analysis with homologs from related *poxviruses*, especially *vaccinia virus*, we highlight both evolutionarily conserved functions and potential unique adaptations in LSDV proteins. This review further identifies critical knowledge gaps in current understanding and proposes promising research directions. We emphasize that integrating multi-omics approaches with structural biology will be essential for advancing our understanding of LSDV pathogenesis and for developing novel preventive and therapeutic strategies against this important animal pathogen.

## 1. Introduction

*Lumpy Skin Disease* (LSD) is an economically significant viral disease, primarily affecting cattle and, to a lesser extent, water buffalo. Clinical manifestations include fever, characteristic skin nodules, emaciation, reduced milk production, infertility, and sometimes death [1,2]. The disease inflicts substantial economic losses due to decreased milk yield, damage to hides, weight loss, abortion, temporary or permanent sterility in bulls, and mortality, alongside significant costs associated with control efforts and trade restrictions [3]. In addition, the financial burden is magnified by vaccination campaigns, movement bans, vector control, and surveillance and compensation schemes, all of which contribute substantially to the total economic impact [4,5]. The causative agent, the *Lumpy Skin Disease Virus* (LSDV), is a double-stranded DNA virus belonging to the genus *Capripoxvirus*. This genus also comprises *sheeppox virus* (SPPV) and *goatpox virus* (GTPV), with which LSDV shares approximately 96–97% genomic sequence identity. Their genomes feature a conserved core set of genes, while variations occur predominantly in the terminal regions involved in host range and immunomodulation. Although antigenically cross-reactive and capable of inducing partial cross-protection, they exhibit distinct host tropisms; LSDV primarily infects cattle, whereas SPPV and GTPV are specific to sheep and goats, respectively [6]. First identified in Zambia in 1929, LSDV was initially confined to sub-Saharan Africa. However, over the past few decades, it has demonstrated a remarkable capacity for geographic expansion. The virus has progressively spread from Africa to the Middle East, and more recently, it has made significant incursions into Asia and Europe, posing a considerable transboundary threat to livestock industries worldwide [7,8]. Its rapid spread underscores its high contagiousness and the challenges in its control, leading to its designation as a notifiable disease by the World Organisation for Animal Health (WOAH) [7]. Recent outbreaks in previously unaffected regions continue to highlight the ongoing global threat posed by LSDV [9].

This review aims to address the existing knowledge gaps through a comprehensive analysis of LSDV-encoded proteins and their host interactions. Specifically, it will systematically characterize the functional roles of LSDV structural/virion proteins, including architecture, core, and membrane components (Section 4: LSDV Virion Architecture and Key Structural Proteins); characterize the functional roles of LSDV proteins throughout the complete viral life cycle, from entry to egress (Section 5: Molecular Mechanisms of LSDV Proteins in the Viral Life Cycle); elucidate the multifaceted immune evasion and immunomodulation strategies employed by LSDV, with particular emphasis on the targeted molecular mechanisms and host pathways; conduct comparative analyses of protein functions and immunoregulatory tactics between LSDV and related poxviruses, notably *vaccinia virus* (VACV) (Section 6: LSDV Immunomodulatory Strategies); and identify critical knowledge gaps and propose impactful future research directions to advance both understanding and control of LSDV pathogenesis (Section 7: Conclusions and Future Perspective).

## 2. Methods

This review employed a narrative synthesis approach to analyze and integrate scientific literature pertaining to the functions of proteins encoded by the *Lumpy Skin Disease Virus* (LSDV), including publications available up to mid-2025. A comprehensive search was performed across PubMed and Web of Science using key terms such as “*Lumpy skin disease virus*”, “LSDV proteins”, “LSDV genome”, “LSDV gene function”, “LSDV immune evasion”, “LSDV virulence factors”, and “VACV comparative genomics”. The initial search yielded 503 records from PubMed and 557 from Web of Science.

The screening process involved a title/abstract review followed by a full-text assessment based on predefined eligibility criteria. Priority was given to peer-reviewed original research articles, with particular emphasis on (1) proteomic and genomic studies of LSDV; (2) loss- or gain-of-function experiments on viral genes; and (3) investigations into host–virus protein interactions. To further support functional inferences, annotation data from authoritative databases including UniProt and NCBI were incorporated.

Through critical appraisal and integration of the available evidence, this review aims to identify current knowledge gaps in LSDV research and suggest key directions for future studies. Although certain systematic review elements were adopted, this study does not claim full compliance with PRISMA guidelines and instead emphasizes thematic synthesis and comparative functional analysis within the *Poxviridae* family. As a narrative review rather than a systematic one, this article was not conducted following a predefined exhaustive search strategy or formal criteria for study inclusion and quality assessment. Consequently, it does not claim comprehensive coverage of the literature nor complete avoidance of bias. The findings presented herein are intended to provide a reasoned synthesis and interpretation of available evidence rather than a definitive quantitative summary. Thus, they should be regarded as exploratory and provisional, and may help inform future, more rigorous systematic reviews.

## 3. Lumpy Skin Disease Virus

LSDV is a double-stranded DNA virus belonging to the genus *Capripoxvirus* within the subfamily *Chordopoxvirinae* of the *Poxviridae* family. Electron microscopic observations reveal that LSDV virions possess highly complex structures, featuring a lipid bilayer envelope encapsulating an internal viral core surrounded by lateral bodies (LBs), collectively forming an enveloped core complex [10]. The LSDV genome contains 156 putative open reading frames (ORFs) spanning approximately 150 kb [11]. The conserved central region encodes proteins essential for viral replication, transcription, and virion assembly, which maintain the fundamental viral replication cycle [1]. The more variable inverted terminal repeats (ITRs) at both genomic ends encode proteins with diverse functions, including host immune modulation, host-range determination, viral replication, and virion morphogenesis [9]. For instance, the LSDV 001/156 gene, located within the inverted terminal repeats (ITRs), is packaged into virions and expressed during the late phase of infection. Experimental evidence indicates that its encoded protein suppresses type I interferon signaling by disrupting IRF3 dimerization. Deletion of this gene attenuates viral virulence in cattle, directly demonstrating the role of an ITR-encoded protein in immune evasion and disease severity [12]. Furthermore, genomic reviews of LSDV have highlighted that the terminal genomic regions adjacent to ITRs are highly variable and often harbor virulence and host-range determinants, further supporting the involvement of ITR-flanking regions in viral adaptation [13,14]. This genomic organization reflects a conserved evolutionary strategy among *poxviruses*; the core genomic region preserves genetic stability of critical functional genes, while the peripheral variable regions enable dynamic adaptation to host immune pressures through rapid evolution.

Beyond the evolutionary flexibility afforded by this genomic architecture, LSDV also possesses the ability to generate novel hybrid virus strains through homologous recombination, in addition to conventional mutation and selection processes. In field conditions, recombination can occur when different LSDV parental strains or vaccine strains co-infect the same host or cell. Studies have provided evidence of recombinant viruses circulating in the wild in regions such as Russia, Indonesia, and Kazakhstan, suspected to be linked to low-quality live vaccines (or mixed-strain vaccines) containing various fragments of the LSDV genome. These recombinant strains incorporate genomic segments from both vaccine and field strains and may exhibit restored or enhanced pathogenicity and transmission capabilities [15,16,17,18]. Furthermore, in a striking finding, a recombinant LSDV strain was shown to outcompete a classical wild-type strain, replicating more rapidly in primary cell cultures and causing more severe disease in cattle [19].

## 4. LSDV Virion Architecture and Key Structural Proteins

*Poxviruses*, including LSDV, exhibit a complex virion architecture. They exist in two main infectious forms: the Intracellular Mature Virus (IMV) and the Extracellular Enveloped Virus (EEV). The IMV form is the most abundant infectious particle and is primarily responsible for host-to-host transmission. It consists of a biconcave or dumbbell-shaped core that houses the viral linear dsDNA genome complexed with various proteins. Flanking this core are two proteinaceous structures known as lateral bodies. This entire core-LB complex is encased by a single lipid bilayer membrane [20]. The EEV form is essentially an IMV particle that has acquired an additional outer lipid envelope derived from host cell membranes, typically the trans-Golgi network or endosomal membranes. EEVs are crucial for cell-to-cell spread within an infected host and for long-range dissemination of the virus, playing a key role in pathogenesis [21].

### 4.1. Lateral Body Proteins

Lateral bodies are unique, amorphous proteinaceous structures found in *poxviruses*, positioned between the core and the IMV membrane. They are thought to contain a variety of proteins, including enzymes and immunomodulators, which are released into the host cell cytoplasm early during infection to help establish a favorable environment for viral replication.

Based on conserved homology analysis with other *poxviruses*, particularly *vaccinia virus* (VACV), LSDV lateral bodies are predicted to harbor several functionally critical proteins; LSDV031, a homolog of VACV F17, is a highly conserved phosphoprotein. In VACV, F17 orchestrates virion assembly and immune evasion by mediating mTOR-dependent degradation of the host cyclic GMP-AMP synthase (cGAS) during late infection [22,23]. LSDV072 is a homolog of VACV H1, a dual-specificity phosphatase that localizes to lateral bodies and participates in viral transcription [24]. Similarly, LSDV053 is a homolog of VACV G4, which also localizes to lateral bodies and contributes to redox regulation during virion morphogenesis [25]. While these functions are predicted for LSDV based on homology, they have not been directly demonstrated. Key aspects, including the precise composition of lateral bodies, their interactions with host proteins, and their specific roles in early infection, remain unknown. As such, these viral components represent crucial targets for understanding early pathogenesis and are promising avenues for new antiviral strategies.

### 4.2. Membrane Proteins (IMV and EEV)

LSDV, like other *poxviruses*, possesses numerous membrane proteins associated with both IMV and EEV forms (Table 1). These proteins are critical for virion structure, cellular attachment, entry, and egress. The IMV form is the most abundant infectious particle and is primarily responsible for host-to-host transmission. It consists of a biconcave or dumbbell-shaped core that houses the viral linear dsDNA genome complexed with various proteins. Flanking this core are two proteinaceous structures known as lateral bodies. This entire core-LB complex is encased by a single lipid bilayer membrane [20]. The EEV form is essentially an IMV particle that has acquired an additional outer lipid envelope derived from host cell membranes, typically the trans-Golgi network or endosomal membranes. EEVs are crucial for cell-to-cell spread within an infected host and for long-range dissemination of the virus, playing a key role in pathogenesis [21].

IMV membrane proteins form the outer surface of the mature virion and play a critical role in initiating infection of new host cells. A well-studied example is LSDV060, the homolog of VACV L1R, a myristoylated membrane protein essential for both virion assembly and cellular entry. In sheeppox virus, which is also a *capripoxvirus*, the L1 homolog has been shown to elicit neutralizing antibodies, confirming its surface exposure and immunogenic characteristics [26]. These findings align with emerging field data, which show that neutralizing antibodies against LSDV develop robustly by about two weeks post-infection [27]. Another key IMV protein, LSDV117, is the homolog of VACV A27L. This protein binds to cell-surface glycosaminoglycans such as heparan sulfate to mediate virion attachment. In VACV, A27 also promotes fusion between the viral envelope and host membranes and contributes to the formation of stable virion architectures [28]. LSDV117 is predicted to fulfill similar dual functions. Additionally, LSDV encodes a range of other IMV membrane proteins homologous to those in VACV, including LSDV064 (L5), LSDV074 (H3), LSDV100 (A9), LSDV104 (A13), LSDV105 (A14), and LSDV109 (A17) [1]. Each contributes distinctly to virion structure or entry mechanisms; for example, H3 (LSDV074) is another surface protein involved in cell binding [29], while A17 (LSDV109) is a reticulon-like protein that promotes membrane curvature [30].

The EEV envelope proteins represent a distinct class of viral components exclusive to the extracellular enveloped virus form, playing pivotal roles in three critical processes: efficient viral particle release from infected cells, cell-to-cell dissemination, and, under certain circumstances, entry into new host cells. While LSDV possesses functional homologs for the majority of characterized VACV EEV-associated proteins, it notably lacks A56 (VACV A56R) and K2 (VACV K2L). In orthopoxviruses such as VACV, the A56/K2 complex on infected cells interacts with the entry-fusion complex (EFC) to suppress cell–cell fusion; deletion of either A56R or K2L leads to extensive syncytia formation [31]. The absence of A56/K2 homologs in LSDV may thus promote enhanced cell–cell fusion and direct spread, and concurrently reflect/drive its restricted host-range and limited cell-type adaptability [13]. LSDV encodes several pivotal EEV proteins essential for virion envelopment, trafficking, and spread. LSDV028, the VACV F13L homolog, is a palmitoylated phospholipase required for membrane curvature during IMV wrapping and subsequent EEV release. Its conservation implies a functionally conserved role in LSDV EEV biogenesis [32]. LSDV122, LSDV123, and LSDV141 are homologs of VACV A33, A34, and B5, respectively. These proteins assemble into a surface complex essential for coordinating EEV trafficking from the endoplasmic reticulum through the trans-Golgi network while also mediating subsequent cell binding and spread functions [33,34]. LSDV126 is the homolog of VACV A36R, a critical adaptor protein on IEVs. It recruits host kinesin motors via interaction with the F12/E2 complex, enabling microtubule-based transport of IEVs to the cell surface for release as EEVs [35]. Deletion of the A36 protein in VACV reduces the transport efficiency of IEVs to the cell surface. Additionally, it abolishes the formation of actin tails, thereby diminishing the propulsive force for the spread of cell-associated enveloped virions (CEVs) on the cell surface [36].

The high degree of conservation of these structural and membrane proteins across *poxviruses* underscores their fundamental roles in the viral life cycle. However, subtle amino acid variations or differences in protein repertoires (like the absence of an A56R homolog in LSDV) could influence aspects like host range, tissue tropism, or interactions with specific host immune components, warranting further comparative functional studies. From a practical standpoint, many of these membrane proteins (LSDV060, LSDV117, LSDV074, LSDV141, etc.) are attractive targets for vaccine development; they are surface-exposed and induce neutralizing antibody responses. In summary, the membrane proteins of LSDV play a pivotal role in viral pathogenicity and immune evasion by facilitating efficient cellular entry and subsequent release of viral particles for further transmission. Yet, despite their clear functional importance and direct exposure to the host immune system, the precise identities of the protective antigens among them remain elusive. This challenge is underscored by the fact that, despite decades of research into live-attenuated *capripoxvirus* vaccines, the identification of precise protective antigens remains a core challenge—viral proteins that confer sterilizing or fully protective immunity. Reflecting this difficulty, Teffera et al. noted that while the *capripoxvirus* genome contains ~156 open reading frames, a targeted screen for membrane-associated, immunogenic proteins with homology to *orthopoxvirus* immunogens yielded only 13 candidates. Crucially, none have been definitively proven to function as a standalone protective antigen in natural hosts like cattle [37].

### 4.3. Core Proteins

The core of an LSDV virion is a tightly organized nucleoprotein complex containing the viral genome and enzymes for early transcription. Core proteins form the interior scaffold that encases and protects the DNA and orchestrates its release once inside a host cell. Many core components are highly conserved across poxviruses and are essential for virion assembly (Table 2).

The major core proteins of LSDV are homologs of VACV core proteins, including A3, A4, A10, and L4, which are often designated by their virion polypeptide names such as P4b and P4a. For example, LSDV094 corresponds to VACV A3L and encodes the P4b protein, a DNA-binding component that contributes to the formation of the core’s palisade layer [38]. A recent study of VACV revealed that P4a (A10) constitutes a major core protein whose trimeric formation not only maintains the poxvirus’s characteristic brick-shaped core but also mediates connections between the core, viral envelope, and lateral bodies while additionally modifying the core wall to enable viral DNA release during infection [39]. It is therefore likely that LSDV101, the homolog of A10, serves a similar function by anchoring the core to viral membranes and lateral bodies. LSDV063 is the homolog of VACV L4R and encodes the VP8 protein, a DNA-binding protein essential for the transcription of early genes [1,40,41]. Another key protein, LSDV095, is the homolog of VACV A4L and is involved in core morphogenesis and DNA retention within the core. Although A4 remains less fully characterized, it is known to interact with other core components during assembly [42,43].

In summary, LSDV’s core proteins create a protective shell for the genome and a platform for the early gene expression that occurs within minutes after infection. Table 2 lists LSDV core proteins alongside their functions and VACV counterparts. As expected, these proteins are among the most conserved between LSDV and other poxviruses, reflecting strong evolutionary constraints; even single amino acid changes in core proteins can be lethal to the virus. Interestingly, some core proteins have moonlighting roles. Understanding these nuances in LSDV is important, as it helps to reveal subtle differences related to its host specificity or virulence in cattle.

## 5. Molecular Mechanisms of LSDV Proteins in the Viral Life Cycle

The replication cycle of LSDV takes place entirely within the cytoplasm. As a result, the virus must encode a wide array of proteins to independently execute DNA replication, mRNA transcription, and virion assembly without relying on the host nucleus. Viral infection advances through a tightly regulated sequence of early, intermediate, and late gene expression, closely synchronized with viral DNA synthesis and the generation of new virions. This section details how specific LSDV proteins contribute to each phase of the life cycle, spanning from host cell entry and genome replication to gene expression, virion assembly, and eventual release.

### 5.1. Proteins Orchestrating Viral Entry

The entry of LSDV into susceptible host cells is a critical first step in establishing infection. Recent studies indicate that LSDV utilizes macropinocytosis as a primary route of entry, a process that is dependent on dynamin and requires a low pH environment for successful uncoating [44]. Following cellular uptake, the viral membrane undergoes pH-dependent fusion with host endosomal membranes, thereby releasing the transcriptionally active viral core into the cytosol.

This fusion event is mediated by a conserved multi-protein assembly known as the Entry-Fusion Complex (EFC). In VACV, the EFC comprises eleven core proteins (A16, A21, A28, F9, G3, G9, H2, J5, L1, L5, and O3) that work in concert to mediate membrane fusion [45]. LSDV encodes homologues for ten of these eleven EFC proteins (Table 3): LSDV118, LSDV113, LSDV108, LSDV024, LSDV059, LSDV052, LSDV073, LSDV070, LSDV064, and LSDV060. The absence of an O3 homolog in the LSDV genome [1] is consistent with its broader loss across several *Chordopoxvirinae* genera, implying functional replacement or adaptation [46]. In VACV, O3 loss is partially compensated by mutations in other EFC transmembrane domains, illustrating the complex’s functional plasticity [47]. Whether a similar compensatory mechanism operates in LSDV remains unknown. Specific EFC components have defined roles. For example, L1 (LSDV060) is a myristoylated membrane protein that functions at the fusion interface [48]. Inhibition of L1 N-myristoylation results in the production of non-infectious virions that are defective in cellular entry [49]. H2 (LSDV073) and A28 (LSDV118) are essential for viral entry and cell–cell fusion [50,51]. A21 (LSDV113) is proposed to sense low-pH conditions and undergo conformational changes that catalyze membrane fusion [52]. The coordination among these proteins is highly intricate and depends on precise stoichiometric balance; even minor deviations in the abundance of individual components can disrupt the fusion process.

Although the overall architecture of the EFC is conserved in LSDV, key aspects such as the stoichiometry, dynamic interactions, and functional contributions of its individual components, particularly given the apparent absence of an O3 homolog, require further experimental validation through structural biology approaches including cryo-EM of the intact complex and targeted functional assays such as reverse genetics. In summary, LSDV enters host cells via a conserved mechanism that involves virion surface protein-mediated attachment, endocytic internalization, and EFC-driven membrane fusion, ultimately delivering the viral core into the cytoplasm. Elucidating this entry pathway not only advances fundamental virological knowledge but also highlights practical therapeutic opportunities, since this stage is vulnerable to neutralization by antibodies or inhibition by entry-targeting compounds.

### 5.2. Proteins Driving Viral Replication

LSDV, like other poxviruses, is distinguished from most DNA viruses by its exclusive cytoplasmic replication strategy. The entire viral life cycle occurs within specialized cytoplasmic structures known as viral factories. Due to this compartmentalization, LSDV does not rely on the host nucleus for transcription or replication and must encode its own machinery for these processes, in addition to factors involved in genome protection and repair. Following the expression of early genes, the viral core enters the cytoplasm and initiates DNA replication. This replication process is complex and involves multiple enzymes. Similar to VACV, LSDV encodes most of the enzymes required for genome replication, including a DNA polymerase, a processivity factor, various nucleic acid metabolic enzymes, and ligases, among others.

LSDV039 serves as the core DNA polymerase in LSDV and is the homolog of VACV E9L. It is a high-fidelity enzyme responsible for genome synthesis. Both E9 in VACV and its counterpart LSDV039 in LSDV are essential for replication [53]. The double mutant L578A+I582A of E9 cannot be rescued in VACV, which confirms the essential role of E9 in viral replication [54]. LSDV039 is predicted to function in conjunction with a heterodimeric processivity factor composed of LSDV112 (homolog of VACV A20) and LSDV082 (homolog of VACV D4). In VACV, the A20-D4 complex acts as a sliding clamp loader that anchors the polymerase to DNA and significantly increases its processivity [1,53]. LSDV also expresses several DNA-binding proteins that support viral replication. LSDV045 (homolog of VACV I3L) encodes a single-stranded DNA-binding phosphoprotein. LSDV083 (homolog of VACV D5R) is predicted to encode an essential DNA-dependent ATPase/helicase involved in processes such as replication initiation [55]. Furthermore, LSDV encodes nucleotide metabolic enzymes that facilitate viral replication. LSDV020 (homolog of VACV F4L) represents the small subunit of ribonucleotide reductase (RNR). It associates with a host- or virus-derived large subunit to convert ribonucleotides into deoxyribonucleotides, thereby supplying essential precursors for DNA synthesis [56]. Additionally, LSDV066 (homolog of VACV J2R) encodes thymidine kinase, which supports nucleotide salvage pathways.

All the above replication proteins are encoded within the central genomic region of LSDV, underscoring their conserved and essential roles. As summarized in Table 4, these LSDV DNA replication genes and their respective functions closely mirror those of VACV, reflecting shared evolutionary adaptations to cytoplasmic replication. Generally, LSDV possesses a complete set of replication machinery that enables autonomous cytoplasmic replication. Nevertheless, targeted investigations into LSDV-specific protein functions and their potential unique regulatory interactions within bovine cells remain necessary to fully elucidate their biological significance.

### 5.3. DNA Integrity and Genome Maintenance

During the replication of their large DNA genomes, poxviruses like LSDV are susceptible to DNA damage and replication errors. To preserve genome integrity and promote genetic recombination, these viruses encode a suite of specialized enzymes. These proteins are essential for repairing DNA breaks and ensuring the accurate distribution of genomic material to progeny virions. Moreover, by counteracting DNA damage, they bolster viral resistance to host-derived antiviral defenses.

A pivotal enzyme is a Fen1-like nuclease, represented in LSDV as LSDV054 (homologous to VACV G5R). Fen1 (flap endonuclease) processes Okazaki flaps and resolves branched DNA intermediates. In VACV, G5 is critical for cleaving concatemeric replication products and for repairing DNA breaks; catalytic inactivation of G5 abolishes genome maturation and impairs break repair [57,58]. LSDV also encodes uracil DNA glycosylase (UDG; LSDV082, homologous to VACV D4R), which removes uracil residues arising from cytosine deamination, thereby preventing G:C to A:T transitions; and dUTPase (LSDV018, VACV F2L homolog), which helps prevent uracil incorporation by depleting the dUTP pool. These two enzymes operate in tandem; dUTPase limits the substrate, and UDG corrects misincorporations. This layered defense enhances replication fidelity, a necessity for large viral genomes. Notably, in VACV, simultaneous disruption of both UDG and dUTPase yields a more severe phenotype than either single mutant under low-repair conditions [59]. Additional maintenance factors encoded by LSDV include LSDV077 (topoisomerase I homolog, VACV H6R) to relieve torsional strain, LSDV114 (Holliday junction resolvase homolog, VACV A22R) to process recombination intermediates, and LSDV133 (DNA ligase homolog, VACV A50R) to seal nicks and complete repair. All these genes are centrally located in the genome and are summarized in Table 4.

The presence of these genes underscores that LSDV, similar to vaccinia virus, utilizes extensive homologous recombination during its replication cycle. This molecular behavior is reflected in characteristic poxviral phenomena such as high-frequency marker exchange and the prevalent formation of genomic concatemers. Functionally, recombination acts not only as a DNA repair pathway but also as a source of genetic diversity. Notably, field studies have identified naturally occurring recombinant LSDV strains, including those involving vaccine-derived genetic material [17]. These observations confirm that the molecular maintenance systems are operative under natural conditions and can facilitate the emergence of novel viral genotypes. Generally, LSDV encodes a robust toolkit for DNA repair and recombination to safeguard its large genome. These enzymes minimize lethal mutations and allow the virus to maintain viability even under attack by host DNA-editing enzymes. They also permit the virus some genetic plasticity, potentially aiding in evolution of virulence. Targeting these proteins, advanced bioinformatics approaches can be employed for virtual screening of potential drug candidates. For instance, Sabbir Zia et al. successfully identified potential inhibitors targeting the LSDV-encoded DNA polymerase protein through computational analysis and molecular docking strategies [60]. This highlights the promising therapeutic potential of targeting these proteins combined with bioinformatics approaches for LSDV treatment development.

### 5.4. Viral Transcription Machinery

LSDV undergoes tightly regulated gene expression during its cytoplasmic replication cycle. Rather than relying on the host nuclear RNA polymerase II, the virus is predicted to encapsulate its own multi-subunit RNA polymerase and associated transcription factors. This self-contained system enables immediate early gene expression upon infection. Uniquely among viruses, poxviruses encode a complete transcription machinery, including enzymes responsible for capping and polyadenylation, which ensures fully autonomous mRNA synthesis.

LSDV possesses an eight-subunit core RNA polymerase that is highly conserved and functionally analogous to that of VACV. The subunits are encoded by genes homologous to VACV RPO147, RPO132, RPO30, RPO35, RPO22, RPO18, RPO19, and RPO7, corresponding to specific LSDV orthologs (list in Table 5). While this polymerase complex carries out viral gene transcription, it depends on stage-specific transcription factors to regulate promoter recognition and temporal expression [61]. The initiation of early transcription is facilitated by the early transcription factors LSDV084 and LSDV098, which specifically recognize promoter sequences and recruit the viral RNA polymerase to synthesize early viral mRNAs [1]. Transition to intermediate gene expression is governed by the viral intermediate transcription factor 3 (VITF-3), encoded by the LSDV099 and LSDV115 genes [62]. Subsequently, late-stage transcription requires three late transcription factors (VLTF-4, VLTF-3, and VLTF-2), corresponding to LSDV076, LSDV092, and LSDV091 gene products, respectively [1]. Of particular significance, VLTF-4 exhibits dual functionality, contributing to both late gene transcriptional regulation and viral DNA replication [63]. LSDV encodes a suite of mRNA processing enzymes that ensure the stability and translatability of viral transcripts in the host cytoplasm. These include a two-subunit capping enzyme (LSDV079 and LSDV089), responsible for 5′ mRNA capping, and a poly(A) polymerase (LSDV032, homologous to VACV E1L) that adds 3′ poly(A) tails. These components operate in a precisely coordinated temporal sequence throughout the viral gene expression cascade, ensuring efficient and stage-specific transcriptional regulation that ultimately promotes viral replication and spread (Table 5). The unique host-independent transcriptional apparatus of poxviruses highlights their replication and transcription-associated proteins as promising targets for antiviral intervention.

In summary, LSDV encodes a complete transcriptional system that enables cytoplasmic takeover of host protein synthesis without nuclear involvement. This self-sufficiency supports poxviral broad cell tropism while also offering potential antiviral targets. Detailed study of LSDV transcription may reveal functionally distinct features compared to VACV, such as cattle-specific promoter adaptations or host-range transcription factors. Currently, understanding of LSDV transcription still relies heavily on extrapolation from VACV, highlighting the need for direct mechanistic studies.

### 5.5. Viral Proteins Involved in Translation

Although LSDV relies on the host’s ribosomes for protein synthesis, it actively manipulates the translational machinery to favor viral gene expression. Poxviruses are notably capable of suppressing host protein synthesis while selectively enhancing the translation of viral mRNAs, particularly during late infection. Multiple LSDV-encoded proteins contribute to this translational reprogramming.

LSDV employs a key strategy of mRNA decapping and subsequent degradation. The virus encodes homologs of the vaccinia virus (VACV) decapping enzymes D9 and D10, designated LSDV086 and LSDV087, respectively. These enzymes catalyze the removal of the 5′ cap from mRNAs, leading to their degradation by cellular exonucleases [64,65]. They preferentially target host and early viral mRNAs, thereby freeing ribosomes for the translation of viral late mRNAs. Poxviruses can also actively modify host ribosomes to enhance viral mRNA translation. In VACV, the serine/threonine kinase B1R phosphorylates the host ribosomal protein RACK1, enhancing translation of viral late mRNAs with 5′ poly(A) leaders [66]. LSDV encodes a conserved homolog (LSDV139), but direct evidence of RACK1 phosphorylation or translational modulation by LSDV has not yet been reported. Additionally, LSDV encodes two key homologs, LSDV034 (VACV E3-like) and LSDV014 (VACV K3-like), which are primarily recognized for their roles in interferon antagonism. Beyond immune evasion, emerging evidence indicates that both proteins also directly enhance viral translation [67]. However, it should be noted that in the case of LSDV these functional roles remain predicted on the basis of homology and are not yet experimentally confirmed.

LSDV ensures that once its mRNAs are synthesized, they dominate cellular translation. As a cytoplasmic virus incapable of exploiting nuclear export or host mRNA processing pathways, it actively subverts host functions through degradation of cellular mRNAs and structural remodeling of ribosomes to favor viral transcripts, thereby maximizing viral protein output. Although reliant on host ribosomes, LSDV proactively reprograms translation to serve viral priorities. This multi-layered disruption of host translation, a recognized hallmark of poxvirus infections, is fully functional in LSDV. Further studies in bovine cells or in vivo systems are necessary to clarify the extent to which LSDV-induced translational shutdown contributes to cytopathic effects or immune evasion in cattle. These questions remain both open and biologically significant.

### 5.6. Proteins Involved in Virion Assembly, Maturation, and Egress

The late stage of the LSDV life cycle consists of the assembly of new virions, the maturation of these virions into infectious particles, and the exit of virions from the host cell. Poxvirus virion assembly is a complex, stepwise process that can be divided into (i) formation of crescent membranes and immature virions (IVs), (ii) maturation of IVs into complete IMVs, and (iii) wrapping of some IMVs to form IEVs and their transport for release as EEVs. Each step involves numerous viral proteins, many acting in concert as multi-protein complexes. LSDV encodes homologs of essentially all the known VACV assembly and egress factors, indicating that its morphogenesis follows the canonical poxvirus pathway.

Membrane biogenesis: Poxviruses synthesize de novo lipid membranes in virus factories, which appear as crescent-shaped open disks that eventually close into spherical immature virions. Several viral proteins are dedicated to this step. LSDV has homologs of VACV A6, A11, H7, L2, and A30.5, which are critical for membrane formation. For example, LSDV097 (A6) is required to localize membranes to assembly sites [68,69,70]. LSDV102 (A11) helps stabilize crescent membranes. Notably, A11 mutants in VACV result in fragile membranes that cannot form virions [71]. LSDV078 (H7) and LSDV061 (L2) are required for proper crescent formation and are non-redundant parts of the membrane assembly machinery [72,73]. In particular, LSDV061/L2 is noted as required for crescents to form at all. LSDV107 (A15) is another interesting one. VACV A15 is involved in regulating membrane assembly, and its absence leads to “empty” immature virions lacking cores; thus, LSDV107 likely plays a similar role in core insertion or DNA packaging [74]. It is evident that orchestrating membrane assembly is a finely tuned process requiring these viral scaffolding proteins. If any of these proteins are absent, assembly can arrest.

Genome packaging and core assembly: As the spherical IV forms, the viral DNA and core proteins must be inserted. The major core protein P4a (encoded by VACV A10L, LSDV101 homolog) is crucial for the correct assembly of viral DNA into the nucleoprotein complex, which is then encapsidated to form immature virions [75]. During maturation, core proteins are cleaved by a viral protease (I7, which is also encoded by LSDV) to form the mature core scaffold [76]. Studies on VACV indicate that proper processing of core proteins and the presence of proteins such as D13 are critical. LSDV090 is the homolog of VACV D13, which forms a honeycomb-like scaffold on the surface of immature virions. D13 acts as a temporary mold that gives shape to the virion; its absence blocks particle formation [77].

Virion maturation: After immature virions (IVs) are fully formed and loaded with DNA, they undergo dramatic internal changes to become infectious IMVs. Key maturation events include specific proteolysis of core proteins by the virus-encoded I7 protease (VACV I7L, LSDV048 homolog); condensation of the viral nucleoprotein complex into an ordered pseudo-hexagonal lattice; and synergistic formation of IMVs’ typical brick shape via folded viral inner membrane, condensed core layer, and surface tubular elements (STEs) [78]. Proteins LSDV057 (VACV G7L) and LSDV111 (VACV A19L) are two key factors required for correct maturation [79,80]. In VACV, G7 and A19 are non-enzymatic proteins; their absence results in viral stalling at the IV stage with abnormal morphology. LSDV057 and LSDV111 presumably ensure that structural transitions (e.g., core wall collapse, DNA condensation) occur properly. Additionally, the redox environment within the virion is regulated during maturation; as previously mentioned, LSDV053 (homolog of VACVG4) likely ensures the correct formation of disulfide bonds in certain late-stage proteins during this process [81,82]. Once mature IMVs are formed, some remain in the cell until cell lysis, while a subset is transported out of the cell. Like VACV, LSDV can exit host cells via two pathways: cell lysis, which releases large quantities of IMVs, and budding as EEVs to enable early dissemination.

Wrapping and egress of EEVs: The formation of an extracellular enveloped virion (EEV) begins when an intracellular mature virion (IMV) becomes wrapped by a double-membrane envelope derived from the trans-Golgi network or late endosomes, forming an intracellular enveloped virion (IEV). This process is mediated by several key viral proteins. Among these, LSDV046 (VACV I5, also termed VP13) has been identified as essential for outer envelope formation and subsequent viral release [32]. While the precise mechanism of I5 remains incompletely understood, it may facilitate membrane envelopment or mediate the fusion of Golgi-derived vesicles around the virion. The EEV glycoprotein complex A33/A34/B5 (LSDV122/123/141), previously discussed for its role in cell entry, also contributes to viral exit. Specifically, A34 and B5 participate directly in the wrapping process; deletion of either protein results in defective IEV formation and impaired EEV release [34]. Thus, LSDV122-123-141 are crucial for generating extracellular virions capable of systemic dissemination via the bloodstream or arthropod vectors.

Following envelopment, intracellular enveloped virions (IEVs) undergo microtubule-dependent transport to the cell periphery. As previously noted, this process involves LSDV126 (A36) and the F12/E2 complex (LSDV027 and LSDV033). Specifically, LSDV027 (VACV F12L) and LSDV033 (VACV E2L) form an internal complex that associates with IEVs and mediates their attachment to kinesin motors through A36 [83,84]. Genetic deletion of either F12 or E2 disrupts proper virion trafficking to the cell surface [85]. Given that LSDV encodes both homologs, it likely employs a comparable transport mechanism.

The final stage of EEV formation occurs when the IEV fuses with the plasma membrane, releasing the enveloped virion while leaving its outer membrane incorporated into the host cell membrane. The resulting EEV retains a single envelope decorated with viral glycoproteins (including A33, A34, and B5), enabling it to initiate new rounds of infection at distant sites. Concurrently, the host cell progressively loses membrane integrity due to continuous virion release and cumulative virus-induced damage, ultimately leading to cell death—typically through lysis, which liberates any remaining IMVs. All corresponding genes are detailed in Table 6.

In summary, LSDV virion morphogenesis follows the well-trodden path of poxviruses, involving an orchestrated interplay of structural proteins and enzymes. Every step—membrane assembly, DNA packaging, core condensation, wrapping, and transport—is a potential choke point if a required protein is missing or inhibited. The high conservation of these processes means knowledge from model poxviruses is directly applicable, yet it also means LSDV is adept at compensating and very robust in assembly. The challenge moving forward is to exploit slight differences or vulnerabilities in LSDV’s assembly process for therapeutic benefit and to validate in LSDV the functions deduced from homology.

## 6. LSDV Immunomodulatory Strategies

A defining aspect of LSDV pathogenesis is its ability to evade or subvert the host immune response. Cattle mount both innate and adaptive immune responses against LSDV, but the virus encodes numerous proteins that interfere with these defenses, enabling it to establish infection and spread. The terminal regions of the LSDV genome (which are more variable) are enriched in genes devoted to immune evasion. In this section, we discuss how LSDV targets the innate immune pathways (interferon responses, NF-κB signaling, apoptosis), the adaptive immune system (T cells, antigen presentation, etc.), and other host defense processes. We adopt a critical perspective, noting where functions are inferred vs. proven, and highlighting important gaps in understanding the bovine context.

### 6.1. Interference with Host Innate Immune Signaling Pathways

The innate immune response is the host’s first line of defense against LSDV. Key components include pattern recognition receptors (PRRs) that detect viral molecules (like viral DNA or dsRNA), leading to the production of interferons (IFNs) and pro-inflammatory cytokines, as well as activation of antiviral pathways such as RNA-dependent protein kinase (PKR). LSDV, like other poxviruses, has evolved multiple proteins that target these pathways at nearly every level to prevent the host cell from mounting an effective antiviral state.

PKR-dependent and independent evasion of translational arrest: PKR serves as a crucial host sensor for viral double-stranded RNA (dsRNA), a common replication byproduct, which triggers PKR dimerization, autophosphorylation, and subsequent phosphorylation of eukaryotic initiation factor 2α (eIF2α), ultimately leading to global translational shutdown to inhibit viral replication. To evade host translational shutdown mediated by PKR, poxvirus encodes several inhibitory proteins: (1) LSDV034—the VACV E3L homolog—is a dsRNA-binding protein that sequesters dsRNA in the cytoplasm, preventing it from activating PKR [86]. (2) LSDV014—the VACV K3L homolog—is a pseudosubstrate that mimics eIF2α, competing for PKR’s kinase activity. Essentially, K3 tricks PKR into binding it instead of eIF2α, but K3 lacks the critical serine that PKR phosphorylates. Thus, PKR’s action is blunted [87,88]. (3) LSDV087—the VACV D10 homolog—while known for mRNA decapping, also contributes to PKR evasion indirectly by reducing the accumulation of dsRNA [89,90]. Another example is LSDV067, a homolog of the VACV C7L family. C7 is a less well-understood host range factor that counteracts host defenses (SAMD9/SAMD9L proteins in mammals), which can inhibit translation independently of PKR. Notably, myxoma virus M062 (a C7L ortholog) binds human SAMD9 to prevent antiviral growth arrest [91]. VACV C7 can rescue replication in cells with functional SAMD9 even if PKR is knocked out, indicating an alternate pathway being blocked [92]. However, the precise molecular mechanism of these LSDV proteins in bovine cells remains to be fully elucidated.

cGAS–STING Suppression Strategies: Beyond PKR evasion, LSDV also disrupts cytosolic DNA sensing pathways. The Cyclic GMP-AMP Synthase (cGAS)–Stimulator of Interferon Genes (STING) pathway serves as a crucial cytosolic DNA sensor that detects poxvirus infection, triggering TBK1-mediated IRF3 phosphorylation and IFN-β production. LSDV counters this through two mechanisms: (1) the LSDV035-VACV E5 homolog, with a recent study showing that VACV E5 causes proteasomal degradation of cGAS [93]. E5 interacts with cGAS and tags it for destruction, thereby preventing sensing of viral DNA [93]. LSDV035 is postulated to have a function similar to that of the vaccinia virus E5 protein, but its specific role still requires experimental validation. (2) In contrast, the inhibitory function of LSDV127 is experimentally supported. It has been shown to bind TBK1, reduce its K63-linked ubiquitination, suppress phosphorylation, and consequently inhibit IFN-β production [94].

NF-κB Pathway Antagonism: In addition to targeting DNA sensing, LSDV employs multiple strategies to suppress NF-κB signaling, a central regulator of inflammatory and immune responses. LSDV encodes (1) Kelch-like proteins (encode by LSDV019/144/151, homologs of VACV F3L/A55R) that serve as substrate adaptors for Cullin-based E3 ubiquitin ligases to degrade/modify NF-κB components like TRAFs and IKKβ [86,95]; (2) A52R-like family proteins (encode by LSDV001/009/136/142/150) that inhibit TLR/IL-1R signaling by targeting adaptor proteins (TRAF6/IRAKs) upstream of IKK activation [96], with LSDV142 specifically disrupting TBK1-IRF3 interaction to suppress type I IFN and likely NF-κB responses [97].

Interferon Effector Antagonism: Finally, LSDV directly interferes with IFN effector mechanisms to ensure viral survival. Poxviruses counteract IFN-mediated antiviral effects (executed through hundreds of ISGs) via specialized proteins—VACV H1 (LSDV072 homolog), a lateral body phosphatase that dephosphorylates STAT1 to impair IFN-γ/α/β signaling while also participating in viral transcription [98,99]. LSDV072 is packaged in virions (lateral bodies) and likely acts early to preempt any initial IFN responses in the infected cell. VACV H1 deletion causes attenuation largely due to unrestrained STAT activation, so LSDV072 is probably a key virulence factor as well. Another interesting factor is LSDV012, an ankyrin-repeat protein. It was found that LSDV012 competitively binds host IFIT1, which is an inhibitor of translation for non-2′-O-methylated viral RNAs, thereby protecting viral mRNAs in a host-specific manner [100].

In summary, LSDV has a layered defense against the host’s innate immune system. It prevents induction of interferons and inflammatory cytokines (via cGAS, STING, TBK1, NF-κB, IRF3 inhibitors), and even if some ISGs are induced, LSDV blocks their action (via STAT1, IFIT1, PKR, etc.). This comprehensive immunomodulation likely allows the virus to replicate largely unchecked for the first critical days post-infection, facilitating virus spread through the skin and beyond. These evasion tactics also mean that clinical signs may be delayed or reduced initially, and by the time the innate immune system catches on, the virus may have spread to secondary sites.

From a research perspective, innate evasion genes are often virulence factors that can be removed to create attenuated viruses. For example, a rational LSDV vaccine might delete LSDV035, 127, 142, 072, etc., to make a virus that triggers interferon and is quickly cleared, yet still immunogenic. The challenge is balancing attenuation with sufficient immunogenicity. Also, there may be redundancy such that removing one gene does not fully cripple the virus because others compensate. It may require multi-gene deletions for a safe, stable vaccine.

### 6.2. LSDV-Encoded Proteins That Subvert Host Adaptive Immunity

To counteract the adaptive immune response, LSDV employs at least two broad strategies: impairing antigen presentation to prevent activation of lymphocytes and directly inhibiting immune cell functions (such as T cell and natural killer (NK) cell activity). These strategies mirror those seen in orthopoxviruses like vaccinia and ectromelia (mousepox), but LSDV has its own versions of key immunomodulatory genes.

Cytotoxic T lymphocytes (CTLs) recognize virally infected cells by viral peptides presented on MHC class I molecules. Many poxviruses encode proteins to downregulate MHC I [101]. For instance, myxoma virus expresses M153, a K3 family member that induces internalization and degradation of MHC I [102,103]. Although LSDV encodes ORF010 (a putative homolog of these PHD/LAP-domain proteins), to date no published experimental evidence demonstrates that ORF010 down-regulates antigen presentation; the hypothesis remains based on sequence homology. Additionally, LSDV encodes a gene LSDV124 (homolog of VACV A35). VACV A35 was shown to reduce the display of antigenic peptides to CD4+ T cells (possibly by disrupting peptide loading or trafficking in antigen-presenting cells) [104,105]. By homology, LSDV124 is predicted to impair bovine MHC II antigen presentation and thereby reduce helper T cell activation and downstream antibody responses, although to date no direct experimental data in the LSDV system have been published. Collectively, such anti-MHC strategies would allow LSDV-infected cells to hide from T cells, especially in the early days post-infection. Beyond direct inhibition, LSDV may additionally subvert antigen presentation through spatial sequestration mechanisms. A case in point is LSDV154, which contains an endoplasmic reticulum (ER) retention motif (-RDEL) structurally homologous to those in MYXV M-T4 and cowpox virus CPXV203 proteins. This motif may mediate MHC-I retention within the ER, thereby effectively blocking antigen presentation [106,107]. Beyond preventing recognition, LSDV encodes factors to suppress immune cells. One such is LSDV135, which is homologous to the poxvirus B22 family. B22 family proteins (examples: MPXV197, ECTV C15, VACV B22) are known virulence factors that broadly dampen T cell responses and even NK cell responses [108,109,110]. As a member of this family, LSDV135 may share similar mechanisms; the specific impact of LSDV135 on bovine T cells and NK cells warrants investigation.

LSDV also encodes molecules that mimic or hijack host immune signaling. LSDV128 is particularly intriguing; it is predicted to be a CD47 mimic. CD47 is a “self” marker on cells that engages SIRPα on macrophages to deliver a “do not eat me” signal, inhibiting phagocytosis [111]. By producing a viral CD47-like protein, LSDV-infected cells might signal to patrolling macrophages or dendritic cells not to engulf them. This could protect infected cells from being cleared, especially in tissues like skin where macrophages attempt to contain infection. The concept has precedent. Myxoma virus expresses a viral CD47 mimic, designated M128, which functionally replicates the natural interaction between host CD47 and SIRPα. This engagement activates the SIRPα-mediated inhibitory signaling pathway, thereby suppressing the host immune response and facilitating viral evasion from clearance [112]. Additionally, LSDV encodes putative virokines and viroceptors including LSDV005 (encoding Interleukin-10 homolog) suppressing inflammation and T cell responses; LSDV006 and LSDV013 (encoding Interleukin-1 receptor like protein) sequestering this pro-inflammatory cytokine; and LSDV011 (encoding G protein-coupled CC chemokine receptor) blocking inflammatory cell recruitment [1,113]. Functional studies confirm the vaccine potential of immunomodulatory gene knockouts. One study showed that ORF005 knockout LSDV Warmbaths variant showed attenuated replication, induced IFN-γ responses, and provided full protection in sheep and goats [114]. After that, Kara et al. demonstrated that ORF005 and ORF008 knockouts in a virulent LSDV protected cattle against challenge, albeit with notable post-vaccinal reactions [115]. Building on this, Chervyakova et al. constructed a multi-gene deletion strain (Atyrau-5BJN(IL18), lacking LSDV005, LSDV008, LSDV066, and LSDV142), which was non-pathogenic, genetically stable, and conferred full protection with neutralizing antibodies in cattle [116]. These findings collectively indicate that deleting specific immunomodulatory genes can yield safe and effective live-attenuated vaccine candidates across susceptible host species.

### 6.3. Modulation of Host Cell Apoptosis and Autophagy

Beyond classical immune signaling, cells also have intrinsic defenses like apoptosis and autophagy that can limit viral replication. Poxviruses often encode proteins that modulate these pathways to keep the host cell alive (at least until the virus finishes replicating) and to prevent destruction of viral components. LSDV is no exception; its genome encodes multiple candidates that likely function to suppress apoptosis and autophagy.

Apoptosis inhibition represents a common strategy employed by viruses, particularly large DNA viruses, which often encode viral Bcl-2 (vBcl-2) homologs to mimic host anti-apoptotic proteins. These viral analogs bind and sequester pro-apoptotic factors, thereby inhibiting programmed cell death. Lumpy Skin Disease Virus (LSDV) encodes at least three putative vBcl-2-like proteins: LSDV004, LSDV042, and LSDV132. Phylogenetic and sequence analyses indicate that these proteins share significant homology with members of the Bcl-2 family. A well-characterized functional analog is the vaccinia virus N1L protein, which binds BH3-only proteins—such as Bid and Bim—and prevents their activation of Bax/Bak, thereby suppressing mitochondrial apoptosis [117]. It is plausible that LSDV004, LSDV042, and LSDV132 operate through a similar mechanism in bovine cells. Supporting this notion, recent in silico structural analysis revealed that LSDV132 adopts a characteristic Bcl-2-like fold and is predicted to exert anti-apoptotic activity [118]. In addition to its potential mitochondrial anti-apoptotic function, LSDV132 was shown to act as a negative regulator of endoplasmic reticulum (ER) stress by suppressing the PERK-CHOP-caspase-12 signaling axis, thereby inhibiting unresolved unfolded protein response-induced apoptosis and further promoting an intracellular environment conducive to viral replication [119]. Additionally, bioinformatic studies suggest that LSDV004 also exhibits structural and functional features reminiscent of Bcl-2 proteins and may represent a promising molecular target for antiviral therapy [120]. Collectively, these findings suggest that LSDV employs multiple vBcl-2 homologs to delay apoptosis in infected cells, thereby facilitating sustained viral replication. LSDV017 encodes a protein homologous to Myxoma virus M11L, a mitochondrial Bcl-2-like apoptosis inhibitor. The Myxoma virus-encoded M11L protein inhibits apoptosis by binding to and preventing the conformational activation of Bax at the mitochondria, thereby blocking mitochondrial outer membrane permeabilization and preserving cell survival during infection [121,122]. Also noteworthy is LSDV131, a member of the superoxide dismutase (SOD) family commonly found in poxviruses. Its ortholog in MYXV, M131R—a copper-zinc SOD homolog—has been shown to suppress mitochondria-dependent apoptosis [123]. Similarly, LSDV131 has been demonstrated to inhibit apoptosis, likely through its antioxidant activity that mitigates oxidative stress and prevents mitochondrial apoptosis, further supporting LSDV’s multi-faceted strategy to suppress host cell death [124].

Autophagy is a process through which cells degrade cytosolic contents, including pathogens, within lysosomes. Many viruses suppress autophagy to avoid degradation or to interfere with its role in antigen presentation. Poxviruses encode SPI-1 (Serine Protease Inhibitor) family proteins, commonly known as serpins, some of which can inhibit apoptosis or autophagy or both. One such protein, LSDV149, is homologous to VACV SPI-1 (B13) and cowpox CrmA. Serpins often function as caspase inhibitors; for instance, CrmA inhibits caspase-1 and caspase-8, thereby blocking extrinsic apoptosis [125]. Similarly, VACV SPI-1 is known to inhibit apoptosis by suppressing caspase activity [126]. Notably, a recent study demonstrated that VACV SPI-1 suppresses autophagy by inhibiting the nuclear export of the host factor FAM111A, which is essential for autophagy induction [127]. Given its homology to VACV SPI-1, LSDV149 is predicted to disrupt cellular apoptosis and autophagy through a comparable mechanism.

The intricate interplay between LSDV-induced ER stress, the mitochondrial apoptosis pathway, and autophagy modulation, and precisely how viral proteins such as LSDV132, LSDV042, LSDV017, and LSDV149 coordinate the cellular life/death decision to maximize viral progeny production, requires further mechanistic elucidation. Notably, the functions of many LSDV-encoded proteins remain uncharacterized. Future studies elucidating the mechanisms of these proteins and evaluating their potential as vaccines or therapeutic targets will provide a critical theoretical foundation for developing novel intervention strategies against viral immune evasion.

## 7. Conclusions and Future Perspectives

A systematic review of the functions of LSDV-encoded proteins reveals that *Lumpy Skin Disease Virus* has evolved a multifaceted molecular arsenal to ensure its survival and dissemination in the face of host immune defenses. The virus’s large genome enables it to encode proteins that orchestrate every stage of the viral replication cycle while simultaneously counteracting nearly all facets of the host immune response. The central genomic region, which is highly conserved among poxviruses, contains the core molecular machinery essential for viral DNA replication, transcription, and virion assembly. This conservation reflects the functional constraint imposed on these fundamental biological processes, which tolerate little evolutionary variation. In contrast, the more genetically variable terminal regions encode an array of host-interacting proteins that modulate the bovine immune response and influence viral virulence. This genomic architecture underscores the evolutionary balance LSDV maintains between stability and adaptability: preserving robust reproductive functionality while continuously innovating immune evasion strategies to overcome evolving host challenges.

**Figure 1:**
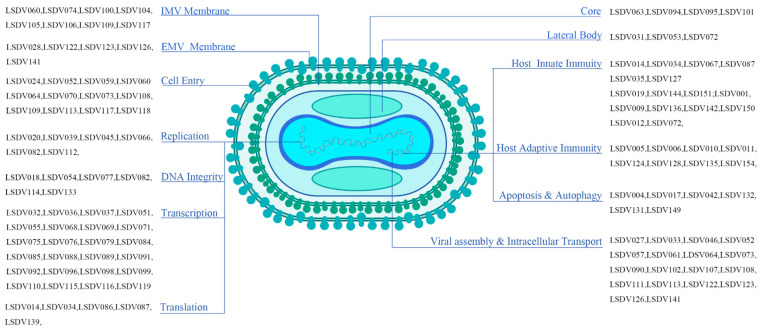
Classification of LSDV genes according to functional roles in structural layers, viral infection cycle, immunomodulation, and apoptosis.

The comprehensive analysis of LSDV proteins reveals a virus that has evolved a multifaceted strategy to ensure efficient replication and immune evasion. The highly conserved central genomic region encodes essential proteins for transcription, translation, and DNA replication, while the variable terminal regions contain genes that modulate host immune responses and determine virulence [1,128]. The structural complexity of LSDV, exemplified by its brick-shaped virion and lateral bodies, reflects an evolutionary adaptation to not only survive but also to manipulate host cellular processes [24,129] (Figure 1).

*Lumpy Skin Disease Virus* (LSDV) employs an extensive array of encoded proteins to coordinate its replication within the host cytoplasm while finely regulating the bovine immune system. Although significant insights have been derived from comparative studies with vaccinia virus, the functional roles of many LSDV proteins and their specific interactions with host components still require direct experimental validation. Several key areas remain poorly understood. (1) Molecular interactions within LSDV protein complexes: further investigation is needed to determine how LSDV proteins assemble into functional complexes such as the entry fusion machinery, replication holoenzyme, and transcriptional apparatus, and how these complexes operate in bovine cellular environments. (2) Comprehensive identification of host factors targeted by LSDV immunomodulators: while several host pathways affected by LSDV have been identified, including interferon signaling, NF-κB activation, and apoptotic processes, the complete set of host proteins that interact with or are modified by LSDV virulence factors has not been systematically characterized. (3) Contribution of immunomodulatory proteins to LSDV virulence and disease outcomes: Substantial evidence implicates proteins such as LSDV010, LSDV154 (MHC modulators), and LSDV135 (a T cell inhibitor) in enhancing viral virulence. Nevertheless, their precise roles in shaping disease progression and clinical manifestations need further elucidation. (4) Molecular mechanisms underlying LSDV host specificity: unlike broad-host-range orthopoxviruses such as vaccinia, LSDV demonstrates a distinct narrow host range confined primarily to cattle. The specific viral factors that dictate this species-specific tropism represent a fundamental unanswered question. These unresolved issues emphasize the necessity for thorough functional analyses to advance our understanding of LSDV–bovine interactions at the molecular level.

Moreover, functional studies aimed at dissecting the precise roles of immune evasion proteins could lead to innovative vaccine strategies that overcome the virus’s ability to subvert host defenses [130,131]. In particular, the modulation of antigen presentation and cytokine signaling by proteins such as LSDV010, LSDV154, and LSDV124 offers promising avenues for therapeutic intervention. Comparative genomic analyses between vaccine strains (e.g., *Neethling*) and wild-type LSDV have identified key mutations in these genes, including frameshifts in LSDV010 and truncations in LSDV154, which attenuate viral immune evasion while retaining immunogenicity [132]. Understanding how these proteins interact with host immune receptors and signaling pathways will be critical for the development of next-generation vaccines that elicit robust and protective immune responses.

The roles of lateral body proteins in early infection events also warrant further investigation. As these proteins are among the first viral factors to be delivered into the host cell, they represent a critical bottleneck in the viral life cycle. Notably, vaccine-associated mutations in lateral body genes have been linked to attenuated virulence. Targeting the functions of lateral body proteins, such as the phosphoprotein F17 and the dual-specificity phosphatase H1 homolog, may provide a means to block the early establishment of infection and improve the efficacy of antiviral therapies [24]. These findings underscore the potential of structure-guided attenuation, whereby targeted mutations are introduced into immune evasion genes to facilitate the development of safer live-attenuated vaccines.

Given the ongoing spread of LSDV into new geographical regions and its significant economic impact on the livestock industry, it is imperative that future studies integrate genomic, proteomic, and immunological data to develop comprehensive control strategies [128]. A multidisciplinary approach that combines molecular virology with advanced bioinformatics and immunology will provide the necessary framework to understand LSDV pathogenesis in depth. Such integrated studies could also offer insights into the evolution of poxviruses more broadly, illuminating how these viruses adapt to overcome host defenses over time [1,129]. Notably, many of the mechanistic insights we reference for LSDV, including viral Bcl-2 homologs, anti-antigen-presentation proteins, and ubiquitin ligase-mediated MHC down-regulation, derive from detailed functional and structural studies in vaccinia virus and other orthopoxviruses. This comparative poxvirus framework provides an established mechanistic blueprint for LSDV research, thereby accelerating hypothesis generation and functional validation in the capripoxvirus context.

In conclusion, the intricate interplay between LSDV proteins and host cell factors underscores the complexity of viral replication and immune evasion. By continuing to dissect these molecular mechanisms, researchers can pave the way for the development of effective antiviral strategies and vaccines. The future of LSDV research lies in the detailed mapping of virus–host interactions.

## Figures and Tables

**Table 1:** LSDV-encoded membrane-associated proteins [1].

LSDV ORF	Predicted Structure and/or Function	VACV ORF
LSDV028	Palmitoylated EEV membrane protein	F13L
LSDV038	Membrane protein	E8R
LSDV060	IMV membrane protein	L1R
LSDV064	Putative membrane protein, required for cell entry	L5R
LSDV070	Late putative membrane protein	J5L
LSDV074	IMV heparin binding surface protein	H3L
LSDV100	IMV membrane protein	A9L
LSDV104	IMV membrane protein	A13L
LSDV105	Phosphorylated IMV membrane protein	A14L
LSDV106	Nonessential hydrophobic IV and IMV membrane protein	A14.5
LSDV109	IMV membrane protein	A17L
LSDV117	IMV surface protein	A27L
LSDV122	EEV membrane phosphoglycoprotein	A33R
LSDV123	EEV glycoprotein	A34R
LSDV126	EEV glycoprotein, TM	A36R
LSDV141	EEV host range protein	B5R

(IMV = intracellular mature virion; EEV = extracellular enveloped virion).

**Table 2:** LSDV-encoded core structural proteins [1].

LSDV ORF	Predicted Structure and/or Function	VACV ORF
LSDV063	DNA-binding virion core protein VP8	L4R
LSDV094	Virion core protein P4b	A3L
LSDV095	Virion core protein, virion morphogenesis	A4L
LSDV101	Virion core protein P4a	A10L

**Table 3:** LSDV gene encoding proteins involved in cell entry.

LSDV ORF	Predicted Structure and/or Function	VACV ORF
LSDV024	S-S bond formation pathway protein	F9L
LSDV052	Component of the entry–fusion complex	G3L
LSDV059	Myristylated protein	G9R
LSDV060	Myristylated IMV envelope protein	L1R
LSDV064	Putative membrane protein, required for cell entry	L5R
LSDV070	Late putative membrane protein	J5L
LSDV073	Component of the entry–fusion complex	H2R
LSDV108	Soluble myristylprotein, required for cell entry	A16L
LSDV109	Phosphorylated IMV membrane protein	A17L
LSDV113	Required for cell entry and fusion	A21L
LSDV117	Fusion protein, virus assembly	A27L
LSDV118	Required for cell entry and fusion	A28L

**Table 4:** LSDV-encoded genes involved in viral DNA replication/DNA Integrity.

LSDV ORF	Predicted Structure and/or Function	VACV ORF
LSDV018	dUTPase	F2L
LSDV020	Ribonucleotide reductase, small subunit,	F4L
LSDV039	DNA polymerase	E9L
LSDV043	DNA-binding virion core protein	I1L
LSDV045	DNA-binding phosphoprotein	I3L
LSDV054	Fen1-like nuclease	G5R
LSDV066	Thymidine kinase	J2R
LSDV077	DNA topoisomerase	H6R
LSDV082	uracil DNA glycosylase	D4R
LSDV083	NTPase; DNA replication	D5R
LSDV112	DNA polymerase processivity factor	A20R
LSDV114	Holliday junction endonuclease	A22R
LSDV133	DNA ligase	A50R
LSDV139	Ser/Thr protein kinase, DNA replication	B1R

**Table 5:** LSDV-encoded genes involved in transcription.

LSDV ORF	Predicted Structure and/or Function	VACV ORF
LSDV032	Poly(A) polymerase PAPL	E1L
LSDV036	RNA polymerase subunit RPO30	E4L
LSDV037	Unidentified role in viral transcription	E6R
LSDV051	Putative transcriptional elongation factor	G2R
LSDV055	RNA polymerase subunit RPO7	G5.5R
LSDV068	Poly(A) polymerase PAPs	J3R
LSDV069	RNA polymerase subunit RPO22	J4R
LSDV071	RNA polymerase subunit RPO147	J6R
LSDV075	RNA polymerase-associated protein	H4L
LSDV076	Late transcription factor VLTF-4	H5R
LSDV079	mRNA capping enzyme, large	D1R
LSDV084	Early transcription factor VETFa1	D6R
LSDV085	RNA polymerase subunit RPO18	D7R
LSDV088	ATPase, nucleoside triphosphate phosphohydrolase-I, homolog of VAC NPH-I	D11L
LSDV089	mRNA capping enzyme, small	D12L
LSDV091	Late gene transcription factor, homolog of VAC VLTF-2	A1L
LSDV092	Putative late transcription factor (VAC VLTF-3)	A2L
LSDV096	RNA polymerase subunit RPO19	A5R
LSDV098	A large subunit of early gene transcription factor, homolog of VAC VETF	A7L
LSDV099	A small subunit of transcription factor, homolog of VAC VITF-3	A8R
LSDV110	DNA helicase; transcriptional elongation	A18R
LSDV115	Large subunit of intermediate gene transcription factor, homolog of VAC VITF-3	A23R
LSDV116	RNA polymerase subunit RPO132	A24R
LSDV119	RNA polymerase subunit RPO35	A29L

**Table 6:** LSDV-encoded genes involved in viral assembly and intracellular transport.

LSDV ORF	Predicted Structure and/or Function	VACV ORF
LSDV027	EEV maturation	F12L
LSDV033	Required for IEV transport, VAC F12/E2 complex associates with kinesin-1	E2L
LSDV046	IMV protein, homolog of VAC VP13	I5L
LSDV052	Required for entry as a component of the entry-Fusion complex	G3L
LSDV057	Virion core protein	G7L
LSDV061	Required for virion assembly, crescent formation	L2R
LSDV064	Putative membrane protein, required for cell entry	L5R
LSDV073	VAC H2R homolog, component of the entry fusion complex	H2R
LSDV090	Rifampin resistance protein, IMV assembly	D13L
LSDV102	Stabilizes membranes during virus assembly	A11R
LSDV107	Absence produces empty immature virions	A15L
LSDV108	Soluble myristylprotein, required for cell entry	A16L
LSDV111	Required for maturation of virus particles	A19L
LSDV113	Required for cell entry and fusion	A21L
LSDV122	EEV membrane phosphoglycoprotein	A33R
LSDV123	EEV glycoprotein	A34R
LSDV126	EEV glycoprotein	A36R
LSDV141	EEV host range protein	B5R

## Data Availability

No new data were created or analyzed in this study.
